# Increased Expression of Histone Proteins during Estrogen-Mediated Cell Proliferation

**DOI:** 10.1289/ehp.0800109

**Published:** 2009-02-07

**Authors:** Zheying Zhu, Robert J. Edwards, Alan R. Boobis

**Affiliations:** Department of Experimental Medicine and Toxicology, Imperial College London, London, United Kingdom

**Keywords:** biomarkers, breast cancer cell lines, estrogenic compounds, histones, protein profiles, SELDI-TOF MS

## Abstract

**Background:**

There is concern about the potential risk posed by compounds with estrogen-like activity present in the environment. As previous studies have shown that combined exposure to such compounds results in dose additivity, it should be possible to assess estrogen exposure with suitable biomarkers of effect.

**Objectives:**

Our goal was to identify candidate protein biomarkers of effect for estrogenic compounds.

**Methods:**

In the search for biomarkers, we assessed the effect of several estrogenic compounds on the expression profile of proteins in breast-derived cell lines varying in their estrogen receptor (ER) phenotype using surface-enhanced laser desorption/ionization time-of-flight mass spectrometry. We identified responsive proteins, after separating them by SDS-polyacrylamide gel electrophoresis, and analyzing the trypsin-digested proteins by tandem mass spectrometry.

**Results:**

The estrogenic compounds 17β-estradiol, genistein, bisphenol A, and endosulfan produced similar protein profile changes in MCF-7 cells (phenotype: ERα^+^/ERβ^+^), but had no effect on MDA-MB-231 (ERα^−^/ERβ^+^), MCF-10F (ERα^−^/ERβ^+^), or MCF-10A (ERα^−^/ERβ^−^) cells. The most responsive proteins in MCF-7 cells were identified as histones H2A, H2B, H3, and H4. Histone levels were not increased in cell lines that showed no proliferative response to estrogens despite their rapid intrinsic growth rate in culture.

**Conclusion:**

Our results indicate that ER-mediated cell proliferation results in up-regulation of core histone proteins.

A rising incidence of endocrine-related diseases in recent decades (Damstra et al. 2002) has led to the suggestion that exposure to endocrine-modulating chemicals (so called endocrine disruptors) is the cause (Colborn et al. 1993; Waring and Harris 2005). There are many such chemicals in the environment that have estrogenic activity, and they are derived from a number of different potential sources. Phytoestrogens, such as genistein (GEN), occur naturally in food as components of dietary plants such as legumes, lentils, chickpeas, soybean, cereals, fruits, and vegetables (Jordan et al. 1985). Industrial contaminants, such as bisphenol A (BPA) and polychlorinated biphenyls, may be present in the atmosphere or as by-products of industrially produced materials such as plastics used as containers for drinks or food (Sheehan 2000). Several organochlorine pesticides, such as endosulfan (EDS), also have estrogenic activity; exposure may occur from household use or from residues in treated food (Safe and Zacharewski 1997). Although EDS is no longer approved for use in many countries, exposure may still be possible through environmental contamination.

To determine the risk to human health, attempts are being made to determine levels of exposure, by measuring either specific compounds or classes of compounds (Dekant and Volkel 2008; Wolff et al. 2007) or by the use of bioassays (Fernandez et al. 2007; Rasmussen et al. 2003) to measure total estrogenic activity present in blood, urine, or adipose tissue (Soto et al. 1997). Although this is useful, it is also informative to determine the biological consequences of exposure to such levels. Consequently, there is much effort to identify suitable biomarkers of effect that can be used for this purpose. To this end, a number of *in vitro* assays have been designed that measure the proliferation of MCF-7 cells (E-screen) (Soto et al. 1995) or binding of estrogenic compounds to the ER through competitive binding assays, or reporter gene assays such as the yeast estrogen cell screening assay (Gutendorf and Westendorf 2001). Several *in vivo* mammalian assays have also been designed. These are based mostly on measurements of female rodent reproductive tissue development. Although it can be argued that some of these tests have physiologic relevance, they appear to perform relatively poorly in terms of quantitation and sensitivity (Ashby 2003).

These approaches may be limited by the complexity of the effects of estrogenic compounds, which appear to have multiple mechanisms of action (Jefferson et al. 2002) that vary for different compounds (Frigo et al. 2002; Naciff et al. 2002). For example, estrogens may act on either or both of the estrogen receptors (ERα and ERβ) as agonists or antagonists and in addition can elicit nonestrogenic effects (Fertuck et al. 2001). Certainly, gene expression studies of both uterine tissue and MCF-7 cells indicate that there is a diverse response to estrogen exposure at the mRNA level (Buterin et al. 2006; Coser et al. 2003; Dip et al. 2008; Frasor et al. 2003). Similarly directed studies have led to a variety of suggested biomarkers of effect by measuring the expression of such genes in cell-based assays (Choi and Jeung 2003). Although changes at the mRNA level are indicative of functional changes, it is more pertinent to study changes in the expression of proteins. Here, to this end, we have employed the use of surface-enhanced laser desorption/ionization time-of-flight mass spectrometry (SELDI-TOF MS) to determine proteomic changes. This technique has been applied to identify potential biomarkers for prognosis, diagnosis, and treatment of human diseases. It has the advantage of being rapid, reproducible, and quantifiable, allows direct sample comparison, and can analyze a wide range of proteins (Petricoin and Liotta 2004). Its use has been explored in classification and evaluation of treatment of breast cancer in patients (Carter et al. 2002; Laronga et al. 2003; Wulfkuhle et al. 2001).

In the present study, four compounds with estrogenic activity were assessed: 17β-estradiol (E_2_) (the natural ligand for ER); GEN (a phytoestrogen); BPA (an industrial contaminant); and EDS (an organochlorine pesticide). Their effect on the protein profiles in four related human breast cell lines that vary in their ER phenotypes was determined. These were human breast cancer cell lines MCF-7 (ERα^+^/ERβ^+^) and MDA-MB-231 (ERα^−^/ERβ^+^) and human breast epithelial cell lines MCF-10F (ERα^−^/ERβ^+^) and MCF-10A (ERα^−^/ERβ^−^), which are well documented for their differential expression of these receptors (Fuqua et al. 1999; Girdler et al. 2001; Hu et al. 1998; Jiang and Jordan 1992; Tong et al. 2002). The objectives of this work were to determine how the proteome of the cell responds to estrogenic compounds, whether different estrogenic chemicals act similarly and, if so, to identify potential common bio-markers of effect.

## Materials and Methods

### Chemicals

We obtained E_2_ (99% purity), GEN (≥ 98% purity), BPA (> 99% purity), and EDS (mixture of forms I and II; 99% purity) from Sigma-Aldrich Company Ltd (Gillingham, UK). Stock solutions of these four estrogenic compounds were prepared as previously described (Zhu et al. 2008b). Unless stated otherwise, we purchased all other analytical grade reagents used in this study from Sigma-Aldrich.

### Cell culture

We obtained MCF-7 and MDA-MB-231 human breast cancer cell lines and MCF-10F and MCF-10A human breast epithelial cell lines from American Type Culture Collection (ATCC; East Greenwich, RI, USA). We cultured MCF-7 and MDA-MB-231 cell lines in RPMI-1640 medium (phenol red-free) supplemented with 10% fetal bovine serum, 100 IU/mL penicillin, and 100 μg/mL streptomycin. MCF-10F and MCF-10A cell lines were cultured in DME/F-12 medium containing 0.04 mM Ca^2+^ and supplemented with 0.37 g/L L-glutamine, 59 mg/L L-leucine, 91 mg/L L-lysine, 17 mg/L L-methionine, 61 mg/L magnesium chloride, 49 mg/L magnesium sulfate, 1.2 g/L sodium bicarbonate, 2.5 μg/mL amphotericin B, 0.5 μg/mL hydrocortisone, 0.1 μg/mL cholera toxin (Merck Biosciences Ltd., Nottingham, UK), 10 μg/mL insulin, 20 ng/mL epidermal growth factor, and 5% horse serum (pretreated using chelex-100 resin; Bio-Rad, Hercules, CA, USA), as suggested by the ATCC. Cell cultures were grown in a humidified atmosphere with 5% CO_2_ in air at 37°C. We harvested cells reaching 60–70% confluence and used them for the following studies.

### Cell proliferation assay (E-screen)

The E-screen was performed as described previously (Zhu et al. 2008b), with some modifications. Briefly, we seeded cells into 96-well microtiter plates at a density of 1 × 10^4^ cells/0.1 mL in each well in phenol red-free RPMI-1640 or DME/F12 medium, as appropriate, before treatment with the estrogenic compounds. Each treatment comprised six replicates. We performed curve fitting of each set of concentration response data and calculations of the lowest effective concentration that produced maximal response (EC_max_), the effective concentration that produced 50% of maximal response (EC_50_), and the highest effective concentration that produced no measurable response (EC_min_) values of each of the estrogenic compounds as described previously (Zhu et al. 2008b). In some instances, we assessed effects on cell viability by trypan blue exclusion, as described previously (Thatcher et al. 2000).

### SELDI-TOF MS analysis of cells

Cells from each of four cell lines were seeded at a density of 1 × 10^5^ cells/mL (2 mL) in 6-well plates (four replicate wells for each treatment) before being exposed to estrogenic compounds. We prepared cell lysates and determined total protein content using the bicinchoninic acid method, as previously described (Zhu et al. 2008b). Analysis by SELDI-TOF MS was performed using CM10 ProteinChip arrays (Bio-Rad) at pH 4.0 with sinapinic acid (Fluka, Steinheim, Germany) as an energy absorbance matrix in a 96-sample bioprocessor format. We analyzed ProteinChip arrays using a Protein Biology System IIc Reader (Bio-Rad), and spectra using Biomarker Wizard software (Bio-Rad). Details of these procedures have been described previously (Zhu et al. 2008b).

### Liquid chromatography-MS/MS

We denatured, reduced, and alkylated the cell lysates and separated them by 1D-SDS PAGE (NuPAGE; Invitrogen, Paisley, UK); we stained the gel with Coomassie blue; excised regions of interest; and decolorized, washed, dehydrated, and digested the proteins with trypsin (sequencing grade, Promega, Southampton, UK) at 37°C for 18 hr, as described previously (Zhu et al. 2008a). Nanoflow LC-MS/MS was performed using an LTQ MS (Thermo-Fisher Scientific, Pittsburgh, PA, USA) to analyze parent ions and data-dependent MS/MS spectra simultaneously. We identified the proteins using Bioworks Browser software, version 3.3 (Thermo-Fisher Scientific) and used SEQUEST to search for matches within the *Homo sapiens* Refseq protein database (National Center for Biotechnology Information 2009).

### Immunoblotting analysis

We separated lysates from treated and untreated cells (3.25 μg protein/lane) and purified calf thymus histones (0.3 μg; Roche, West Sussex, UK) by SDS-PAGE. Histones were then transferred onto nitrocellulose filters; incubated with antibodies against histones H3, H4 (Santa Cruz Biotechnology Inc, Santa Cruz, CA, USA), or H2B (Watson et al. 1995); and detected with goat anti-rabbit peroxidase and electrochemiluminiscence (ECL; GE Healthcare, Amersham, UK) as described previously (Edwards 2006). The relative intensity of the immunoreactive bands was determined by densitometry using an Image Station 440CF and 1D Image Analysis software, version 3.5 (Kodak, Rochester, NY, USA).

## Results

### Concentration effect analysis of cell proliferation

**Figure 1:**
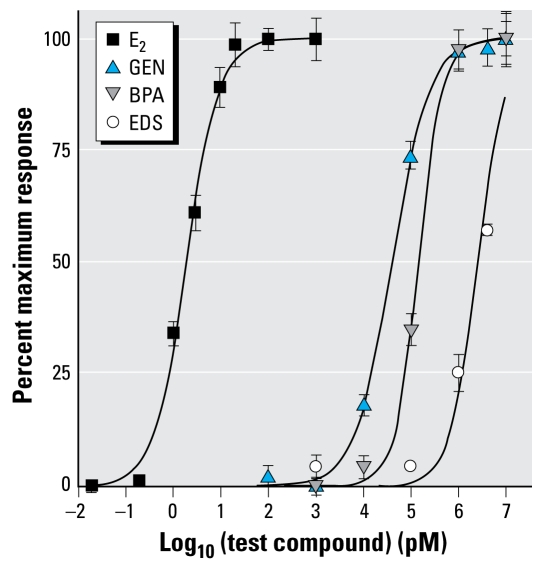
Concentration–effect curves for four estrogenic compounds on the proliferation rate of MCF-7 cells. Microtiter-plate wells were seeded with 10,000 MCF-7 cells per well (0.1 mL) and cultured for 6 days in the presence of E_2_, GEN, BPA, EDS, or vehicle alone. After 6 days, we estimated the relative cell number as described in the text and expressed them as a percentage of the maximal response of E_2_. Each point represents the mean ± SE of six determinations, and data were fitted to sigmoid concentration–effect curves.

We measured the growth of the four cell lines. In the absence of any estrogen, MCF-7 cells grew relatively slowly compared with the other three cell lines (Table 1). However, in the presence of E_2_, the growth rate of MCF-7 cells increased 4-fold relative to control (Table 1). This effect was concentration dependent and characterized by an EC_50_ of 2 pM and EC_max_ of 20 pM (Figure 1), whereas none of the other three cell lines were affected by E_2_ at concentrations up to 2 μM (Table 1). The growth rate of MCF-7 cells also increased 4-fold by treatment with GEN and BPA and 3-fold by EDS relative to control (Table 1). However, GEN and BPA were more than four orders of magnitude less potent and EDS was six orders of magnitude less potent than E_2_. Both GEN and BPA had EC_max_ values of 1 μM (Figure 1), although the maximum responses were similar to that found for E_2_. A full concentration effect curve for EDS was not possible, as the compound was cytotoxic above 10 μM; this compound was similarly cytotoxic to MCF-10F cells but not the other two cell lines. GEN, BPA, and EDS did not cause MDA-MB-231, MCF-10F, or MCF-10A to increase their growth rate (Table 1).

### SELDI-TOF MS protein profiles

**Figure 2:**
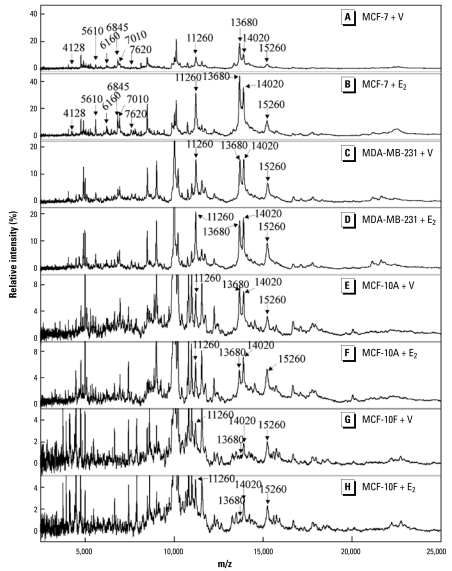
Protein profiles of various cell lines varying in their ER phenotype. SELDI-TOF MS analysis was performed on (*A*) vehicle-treated and (*B*) 20 pM E_2_-treated MCF-7 cells; (*C*) vehicle-treated and (*D*) 20 pM E_2_-treated MDA-MB-231 cells; (*E*) vehicle-treated and (*F*) 20 pM E_2_-treated MCF-10A cells; and (*G*) vehicle-treated and (*H*) 20 pM E_2_-treated MCF-10F cells. A number of protein ions identified as possible biomarkers in MCF-7 cells were also prominent in the spectra of the other cell lines and are indicated by arrows. In each case, treatment of cells with 1 μM GEN, 1 μM BPA, and 10 μM EDS produced similar profiles to those obtained after treatment with 20 pM E_2_.

Protein profiling by SELDI-TOF MS was performed on MCF-7 cells treated separately with 20 pM E_2_, 1 μM GEN, 1 μM BPA, and 10 μM EDS. We used the concentrations that produced a maximal proliferative response in the cells. Each of the test compounds produced a change in the protein profile of the cells, and the changes were similar for all the test compounds, as described previously (Zhu et al. 2008b). In all, from approximately 100 peaks detected, we found 12 protein ions with *m/z* values of 4,128, 5,610, 6,160, 6,845, 7,010, 7,620, 11,260, 11,426, 11,680, 13,680, 14,020, and 15,260 that increased in intensity with treatment with each of the four estrogenic compounds (Figure 2). Other proteins detected by SELDI-TOF MS did not vary significantly between the untreated and treated groups. Among the 12 responsive ions, 8 appeared to be four pairs of doubly charged and singly charged ions from the same proteins, that is, *m/z* 5,610 and 11,260; *m/z* 6,845 and 13,680; *m/z* 7,010 and 14,020; and *m/z* 7,620 and 15,260.

Subsequently, MCF-7 cells were treated with a series of different concentrations of E_2_, GEN, BPA, and EDS to examine levels of these protein ions. Changes in the levels of these eight ions were concentration dependent with each of the four estrogenic compounds examined [see Supplemental Material, Figure 1 (available online at http://www.ehponline.org/members/2009/0800109/suppl.pdf)]; EC_50_ values determined from these data showed that the response to the compounds was similar to that assessed by measurement of cell proliferation (Table 2).

SELDI-TOF MS protein profiles were also obtained using the other three cell lines after treatment with 20 pM E_2_, 1 μM BPA, 1 μM GEN, 10 μM EDS, and vehicle. Although spectra containing numerous peaks were obtained with each of the cell lines, no changes in the protein profiles, or in any individual peak, were found after treatment with E_2_ (Figures 2C 2H) or any of the other compounds tested. Interestingly, although the spectra obtained for the different cell lines varied somewhat, ions with *m/z* 11,260, 13,680, 14,020, and 15,260 identified as potential biomarkers in treated MCF-7 cells were also present in these cell lines (Figure 2).

### Identification of responsive protein ions

**Figure 3:**
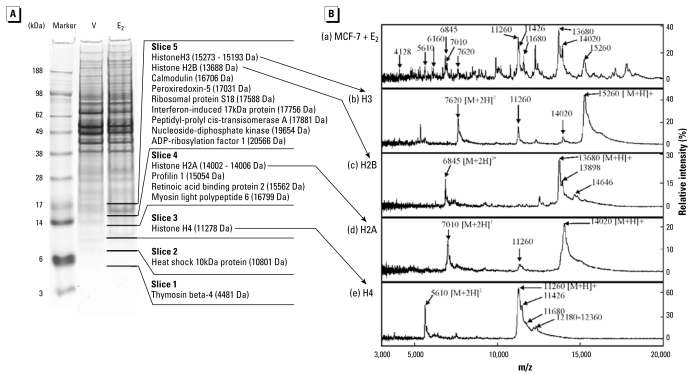
Identification of responsive protein ions. (*A*) Coomassie blue–stained SDS-polyacrylamide gel of whole-cell protein extracts of MCF-7 cells treated with 20 pM E_2_ and vehicle (V). Molecular weight markers are shown on the left-hand side. The lane containing the E_2_-treated cells was divided as shown, and the proteins in each slice of the gel were digested with trypsin in preparation for analysis by LC-MS/MS. The identified proteins found in each slice of the gel are listed along with the predicted molecular weight of each protein. (*B*) Comparison of SELDI-TOF mass spectra of estrogen-responsive protein ions in MCF-7 cells and purified histones. (*a*) 20 pM E_2_-treated MCF-7 cells. The 12 estrogen-responsive protein ions identified are indicated. Purified preparations of calf thymus histones were analyzed under similar conditions with 0.1 μg each of histones (*b*) H3, (*c*) H2B, (*d*) H2A, and (*e*) H4. The major ions evident in each spectrum are indicated along with their likely charge state.

Whole-cell preparations of MCF-7 cells treated with either 20 pM E_2_ or vehicle were subjected to 1D SDS-PAGE. The stained gel shows that the overall protein compositions of the two preparations were similar except for an increase in the intensity of bands of approximately 12 and 17 kDa (Figure 3). The lane containing the E_2_-treated MCF-7 cell proteins was sliced into five sections over the region equivalent to 5–20 kDa corresponding to the likely masses of the proteins detected by SELDI-TOF MS and including bands with increased intensity. Each slice was subjected to trypsin digestion to hydrolyze the proteins into peptides, and then these were analyzed by LC-MS/MS.

Proteins identified on the basis of at least four detected peptides are listed in Figure 3 [for further details see Supplemental Material, Table 1 (available online at http://www.ehponline.org/members/2009/0800109/suppl.pdf)]. All of the core histone proteins were represented (i.e., histones H2A, H2B, H3, and H4). Histone H4 was the only protein identified in slice 3 of the gel, equivalent to the 12 kDa band. Histone H2A occurred in slice 4 along with three other proteins. Both histones H2B and H3, as well as seven other proteins, were detected in slice 5. Protein masses determined by SELDI-TOF MS were accurate to within 0.3%. The masses of each of the core histones were within this error of measurement. None of the other proteins identified by LC-MS/MS had masses within 0.3% of the major peaks in the SELDI-TOF MS spectra [Figure 3; see Supplemental Material, Table 1 (available online at http://www.ehponline.org/members/2009/0800109/suppl.pdf)]. Additional analyses were performed with the same samples on cation-exchange chips at pH 7 and 9 (data not shown). The intensities of the protein ions corresponding to the ions of interest were similar at pH 7 to those obtained at pH 4. They were also clearly detectable at pH 9, albeit with slightly reduced intensity, suggesting that the ions were from highly basic proteins. Histones were the most basic proteins detected in the SDS-PAGE gel [see Supplemental Material, Table 1 (available online at http://www.ehponline.org/members/2009/0800109/suppl.pdf)], further supporting their identification.

To investigate the identity of these proteins further, purified preparations of the core histones were obtained and analyzed by SELDI-TOF MS under the same conditions as those used to analyze the MCF-7 cell preparations (Figure 3). The histones used were from calf thymus; the sequences of human histones are identical or extremely similar to bovine histones (Marino-Ramirez et al. 2006). All of the core histones were readily detected and each was detected as both singly charged and doubly charged species, that is, [M+H]^+^ and [M+2H]^2+^ ions, respectively. In each case, the singly charged ion was more intense than the respective doubly charged ion. All of the ions corresponded to peaks detected using E_2_-treated MCF-7 cells (Figure 3).

Some additional peaks were detected in the spectra of each of the purified histones. These included slightly higher mass species, most evident as shoulders on the main [M+H]^+^ peaks, but also apparent as smaller [M+2H]^2+^ peaks. This was particularly noticeable in the spectra of histones H4 and H2B. For histone H4, ions with *m/z* 11,426 and *m/z* 11,680 were present as overlapping peaks forming a shoulder of the main *m/z* 11,260 ion. Singly and doubly charged species of all of these ions appeared to be present in the spectra of MCF-7 cells. Also, a collection of small peaks was present in the spectrum of histone H4 with *m/z* 12,180–12,360. Ions with *m/z* 6,160 and *m/z* 4128 apparent in the MCF-7 cell spectra may represent doubly and triply charged species of this protein. A similar distribution of peaks was also seen in the spectra of histone H2B, in this case with an ion *m/z* 13,898 forming a shoulder of the main *m/z* 13,680 peak and several smaller peaks such as those with *m/z* 14,646. Although no distinct separate peaks were identified in the corresponding regions of the spectra of histones H2A and H3, the shapes of these peaks were distorted toward higher masses, suggesting that species with increased masses were also present in these preparations. These species probably represent post-translational modifications of the proteins, for example, by acetylation, methylation, phosphorylation, which occur commonly in histones, as well as variant forms of the histones. Such species were also apparent in the cell preparations, but were difficult to quantify as they did not form discrete peaks. Thus, the identity of all 12 of the responsive protein ions could be ascribed to histones or their posttranslationally modified forms.

### Western blotting of histones

Up-regulation of histones H2B, H3, and H4 in MCF-7 cells treated with E_2_ was demonstrated in a previous study by immunoblotting (Zhu et al. 2008a). These effects are compared here with those of GEN, BPA, and EDS (Table 3). All four compounds had similar effects on histone protein levels. Results were consistent with those obtained using SELDI-TOF MS (Table 3).

Comparative levels of histone H2B were determined in all four cell lines used here. Constitutive levels in MDA-MB-231 cells were similar to those in MCF-7 cells, whereas levels in MCF-10A and MCF-10F cells were only about 50% of those in MCF-7 cells (Table 4). Treatment with E_2_ affected H2B expression only in MCF-7 cells; levels were unchanged in the other three cell lines (Table 4).

## Discussion

The ability of E_2_ to stimulate the proliferation of MCF-7 cells is well established (Katzenellenbogen et al. 1987). This is accompanied by a number of cellular changes that have been investigated most comprehensively at the level of gene expression (Buterin et al. 2006; Frasor et al. 2003), although a number of alterations at the protein level have also been demonstrated (Zhu et al. 2008a). Here, we investigated the effect of E_2_ on the whole-cell protein profile using SELDI-TOF MS. It was evident that treatment with E_2_ had a profound upregulatory effect on a number of protein ions detectable by this technique. The minimum concentration of E_2_ that produced a maximal response of the protein ions in MCF-7 cells was 20 pM, and this is similar to that determined using the E-Screen assay on these cells. This value for E-Screen is consistent with that found in several previous studies, which report similar EC_max_ values for E_2_ in the range of 10–20 pM (Soto et al. 1995; Suzuki et al. 2001; Villalobos et al. 1995), although a higher value of 80–100 pM was reported by Coser et al., and a value in the range of 50–200 pM can be deduced from the data shown in Rajapakse et al. (2004). Albeit less potent than E_2_, GEN, BPA, and EDS also increased the proliferation rate of MCF-7 cells, as found in previous studies (Soto et al. 1994; Wang et al. 1996; Zhu et al. 2008b) and resulted in protein profile changes similar to those produced by E_2_. E_2_, GEN, and BPA have previously been reported to produce similar effects on the expression of a number of genes in both MCF-7 cells and the developing female rat reproductive system (Buterin et al. 2006; Dip et al. 2008; Naciff et al. 2002). All the other cell lines studied here lacked expression of ERα, and none of them showed changes in either proliferation rate or in protein profile after treatment with any of the estrogenic compounds. Thus, these data are consistent with the concept that ERα (or ERα/ERβ heterodimer) in MCF-7 cells plays a controlling role in the response to estrogenic compounds (Ter Veld et al. 2006). It was apparent that ERβ alone did not facilitate any response, at least for the compounds examined here. In other studies (Zhu et al. 2008b), we have shown that none of the compounds affected the response to others in MCF-7 cells, suggesting that ERα response is not modified by any effect on ERβ. GEN and BPA appear to bind more strongly to ERβ than ERα (Kuiper et al. 1997) and so might exert a greater effect via ERβ. It is possible that compounds with a greater affiity for ERβ may produce effects other than those found here.

The principal feature of the SELDI-TOF MS protein profile changes in MCF-7 cells after estrogen treatment was an increase in the levels of core histone proteins. Histone proteins are a group of relatively small basic proteins with masses in the range of 11–15 kDa and isoelectric points (p*I*s) > 10. The ready detection of such proteins by SELDI-TOF MS is consistent with the conditions used. Basic proteins would be expected to bind strongly to the cation exchange interactive surface at pH 4, and proteins in the range of 5–20 kDa are particularly well suited to detection by SELDI-TOF MS (Petricoin and Liotta 2004). Up-regulation of these core histones is also supported by immunodetection and a label-free quantitative LC-MS/MS approach used to examine the differentially expressed proteins in E_2_-treated MCF-7 cells (Zhu et al. 2008a). Previously, microarray and RT-PCR analysis of MCF-7 cells treated with 100 pM E_2_ for 24 hr was reported to cause up-regulation of his-tone H2A genes (members X and Z), although in the same study a decrease in the expression of histone 1 H2ac and H2BE genes was also reported (Buterin et al. 2006). In contrast, no variation in mRNA levels of histones in the developing female reproductive system of rats treated with 17α-ethynyl estradiol (Naciff et al. 2002) was reported. Histone proteins are core components of nucleosomes. A nucleosome is composed of an octamer of histone proteins comprising two molecules each of histones H2A, H2B, H3, and H4. Nucleosomes provide a structure around which DNA is coiled and allow formation of compacted higher ordered structures, that is, chromatin. DNA accessibility is regulated via a complex set of post-translational modifications of the highly conserved core histones H2A, H2B, H3, and H4 such as acetylation, methylation, phosphorylation, ubiquitination, and ADP-ribosylation (Bartova et al. 2008; Bonisch et al. 2008).

In this way, histones can regulate gene expression, as well as cell growth and proliferation (Jenuwein and Allis 2001). Up-regulation of all the core histones H2A, H2B, H3, and H4 to the same extent as found in this present study, may represent an increase in the chromatin content of cells and appears consistent with the mitogenic effect of E_2_ on MCF-7 cells.

It was evident that changes in the levels of core histones mirrored the degree of proliferation induced by each of the four estrogenic compounds. Concentration–effect curves produced on the basis of relative intensity of ions representing each of the singly charged histones were quantitatively similar to those for cell proliferation. This might suggest that histone protein levels are simply indirect markers of cell proliferation; indeed, up-regulation of histone mRNA levels as a cell proliferation marker is well documented (Bosch et al. 1993; Muskhelishvili et al. 2003; Slowinski et al. 2005). However, although the cell lines investigated here were insensitive to E_2_, GEN, BPA, and EDS, they still grew rapidly while expressing relatively low levels of histone proteins. In fact, the rate of growth of MCF-10F cells exceeded that of E_2_-stimulated MCF-7 cells, and yet among the cell lines examined, this one contained the lowest levels of histone proteins. Hence, histone protein levels do not appear to reflect the rate of cell proliferation as do mRNA levels. On the basis of these results, it would appear possible that core histone proteins might serve as biomarkers of ER-mediated cell proliferation. This hypothesis warrants further investigation using a variety of other estrogen-sensitive and -insensitive cells, including those of primary origin.

In summary, using proteomic profiling by SELDI-TOF MS, it was possible to identify a number of protein ions that varied on treatment of MCF-7 cells with a variety of structurally diverse estrogenic compounds. These changes reflected mainly up-regulation of histones H2A, H2B, H3, and H4 after ERα activation. It is possible that up-regulation of core histones is selectively indicative of ER-mediated cell proliferation.

## Figures and Tables

**Table 1:** Relative rates of proliferation of various cell lines and the effect of estrogenic compounds: relative increase in MMT absorbance per day (mean ± SE).

	MCF-7	MDA-MB-231	MCF-10A	MCF-10F
Control	0.10 ± 0.01	0.35 ± 0.04	0.41 ± 0.02	0.55 ± 0.05
100 pM E_2_	0.43 ± 0.04**	0.34 ± 0.01	0.40 ± 0.01	0.55 ± 0.03
2 μM E_2_	0.45 ± 0.05**	0.35 ± 0.01	0.41 ± 0.01	0.53 ± 0.03
1 μM GEN	0.45 ± 0.04**	0.34 ± 0.01	0.43 ± 0.01	0.59 ± 0.01
1 μM BPA	0.43 ± 0.05**	0.35 ± 0.01	0.41 ± 0.01	0.56 ± 0.02
10 μM EDS	0.32 ± 0.01**	0.36 ± 0.01	0.40 ± 0.01	0.34 ± 0.01*

We seeded cells into 96-well plates, and the relative rates of linear growth in the absence or presence of estrogenic compounds were determined using the E-screen assay at daily intervals. Results are expressed as relative absorbance values per day in culture. MCF-7 and MDA-MB-231 cells were cultured for 6 days, MCF-10A cells for 5 days, and MCF-10F cells for 4 days according to the characteristics of each of the four cell lines (*n* = 3 in each case).

* *p* < 0.05 and

** *p* < 0.01, compared with control (vehicle-treated) by Student’s *t*-test.

**Table 2:** Comparison of EC_50_ (mean ± SE) values for estrogenic compounds on MCF-7 cells determined by measurement of cell proliferation and SELDI-TOF MS protein ion intensity.

Parameter	E_2_ (pM)	GEN (nM)	BPA (nM)	EDS (μM)
Cell proliferation	2.4 ± 0.7	40.7 ± 1.7	158 ± 51	2.5 ± 0.2
*m/z* 5,610 ion	2.6 ± 0.3	46.9 ± 7.0	155 ± 19	2.0 ± 0.1
*m/z* 6,845 ion	2.2 ± 0.3	45.1 ± 7.7	143 ± 14	2.4 ± 0.4
*m/z* 7,010 ion	2.3 ± 0.5	52.9 ± 7.8	149 ± 37	2.3 ± 0.4
*m/z* 7,620 ion	2.6 ± 0.3	47.6 ± 7.0	139 ± 23	1.9 ± 0.4
*m/z* 11,260 ion	2.3 ± 0.7	54.5 ± 15.3	144 ± 33	1.8 ± 0.4
*m/z* 13,680 ion	2.2 ± 0.6	52.8 ± 14.6	155 ± 39	2.1 ± 0.6
*m/z* 14,020 ion	2.6 ± 1.0	52.8 ± 3.0	176 ± 31	2.1 ± 0.2
*m/z* 15,260 ion	2.5 ± 0.3	42.0 ± 5.0	176 ± 20	2.0 ± 0.2

EC_50_ values were determined from concentration–effect data by curve fitting using a four-parameter sigmoidal model by nonlinear regression. SEs were calculated from replicate experiments (*n* = 3). No significant differences between the values obtained by the cell proliferation method or the intensities of the ions determined by SELDI-TOF MS were found for any of the compounds.

a *p* > 0.05 in all cases by Student’s *t*-test.

**Table 3:** Relative levels of histone proteins in MCF-7 cells after treatment with estrogenic compounds.

	Relative histone level (mean ± SE, *n* = 4)						
	H2A	H2B		H3		H4	
Treatment	SELDI	SELDI	IB	SELDI	IB	SELDI	IB
E_2_	2.3 ± 0.6	2.1 ± 0.3	2.3 ± 0.1	3.1 ± 0.3	2.9 ± 0.2	2.1 ± 0.4	2.6 ± 0.1
GEN	2.2 ± 0.3	2.2 ± 0.6	2.2 ± 0.2	2.5 ± 0.4	2.6 ± 0.3	2.1 ± 0.4	2.8 ± 0.3
BPA	2.1 ± 0.5	2.1 ± 0.1	2.2 ± 0.1	2.6 ± 0.3	2.2 ± 0.2	2.1 ± 0.2	2.7 ± 0.4
EDS	2.3 ± 0.2	2.0 ± 0.5	2.4 ± 0.1	2.4 ± 0.2	2.8 ± 0.2	2.2 ± 0.6	2.6 ± 0.2

We treated cells with 20 pM E_2_, 1 μM GEN, 1 μM BPA, or 10 μM EDS for 6 days, and compared the levels of histones with those of vehicle-treated cells by SELDI-TOF MS (SELDI) and immunoblotting (IB). Immunoblotting data was not obtained for histone H2A, as no suitable antibody was available. Both methods performed similarly; there were no statistically significant differences between levels of histones determined by SELDI-TOF MS or immunoblotting. *p* > 0.05 in all cases by Student’s *t*-test.

**Table 4:** Comparative levels of histone H2B in each of the four breast-derived cell lines.

	Relative histone H2B level	
Cell line	Control cells	E_2_-treated cells
MCF-7	1.00 ± 0.06	2.30 ± 0.11**
MDA-MB-231	1.18 ± 0.10	1.22 ± 0.02
MCF-10A	0.52 ± 0.02**	0.51 ± 0.03
MCF-10F	0.41 ± 0.05**	0.43 ± 0.07

NS, not significant. The levels relative to total cell protein and normalized to those measured in vehicle-treated MCF-7 cells were determined by immunoblotting in each of control (vehicle-treated) cells and cells treated with 20 pM E_2_.

** *p* < 0.01 compared with control (vehicle-treated) MCF-7 cells by Student’s *t*-test.
